# Editorial: Recent Advances in Micro-Nanostructured Optoelectronic Devices

**DOI:** 10.3389/fchem.2022.920807

**Published:** 2022-06-02

**Authors:** Yue-Feng Liu, Xiu-Min Gao, Yun-Fei Li

**Affiliations:** ^1^ State Key Laboratory of Integrated Optoelectronics, College of Electronic Science and Engineering, Jilin University, Changchun, China; ^2^ Center for Advanced Laser Technology, Hebei University of Technology, Tianjin, China; ^3^ Hebei Key Laboratory of Advanced Laser Technology and Equipment, Tianjin, China

**Keywords:** optoelectronic (OE) devices, micro-nano structure, light emitting device (LED), laser, sensor, perovskite

Optoelectronic devices, including lasers, light-emitting diodes (LED), optical detectors and solar cells, have gained substantial attention in scientific research and been widely used in military and national economy fields such as laser detection and measurement, display and solid-state lighting, optical communications and environmental monitoring, and renewable energy sources (Bonaccorso et al., 2010; Lopez-Sanchez et al., 2013; Cao and Yan, 2021; Ye et al., 2021; Mao et al., 2021). The performance of optoelectronic devices needs to be continuously optimized towards high efficiency, small size, and low power consumption to meet the increasing demand of consumers. However, further improvement in performance remains a daunting challenge due to limited light extraction or absorption in conventional device architectures. Inspired by the organisms in nature, a growing number of researchers have demonstrated that micro-nanostructures could attribute unique optical, electrical, and mechanical properties to optoelectronic devices thus serving as one of the most promising solutions to this problem (Bi et al., 2013; Feng et al., 2017; Fusella et al., 2020; Linic et al., 2021). By designing and optimizing the construction in optoelectronic devices, the various micro-nanostructures can be acquired to realize corresponding efficient light manipulation effect such as enhancing light scattering and reducing light reflection, utilizing resonant plasmonic structures to improve light extraction or light absorption, adopting specific resonator geometries to achieve optical feedback, using photonic crystal to guide light propagation path and designing optical metasurfaces to control radiation properties directly at the source level (Choi et al., 2019; Joo et al., 2020; Liu et al., 2020; Zhou et al., 2020; Fu et al., 2021).

To present these inspiring developments, we launched a Research Topic in Frontiers in Chemistry entitled “*Recent Advances in Micro-Nanostructured Optoelectronic Devices*.” This Research Topic covers fabrication, light management mechanisms of micro-nanostructures in optoelectronic devices, and their emerging applications in display and lighting, solar energy harvesting, telecommunication, light sensing and detection, and other fields, including seven minireviews and three original research articles contributed from 51 researchers.

The controllable fabrication of micro-nanostructures is a crucial condition to construct efficient micro-nanostructured optoelectronic devices. With the emergence of various kinds of micro-nanostructures manufacturing technologies, laser fabrication technologies can be used to fabricate micro-nanostructures by the interaction between lasers and materials, which show high efficiency, high precision, and low thermal effect, proving to be a competitive manufacturing method. To this end, a minireview article was delivered to address recent advances in micro-nanostructures fabricated by laser technologies for optoelectronic devices (Yi et al.), which covers both typical light trapping mechanism and surface plasmon-polariton of micro-nanostructures and outlines the typical applications such as photodetectors, photovoltaic cells, organic light-emitting devices. Among kinds of structures, grating structures have demonstrated the superior light trapping properties, coupled with simple fabrication process and easy adjusting of size and morph, which make it widely used optical management structures. Bai et al. introduced 1D and 2D grating structures into the hole injection layer of organic light emitting diode (OLED) to couple the power loss trapped in devices. As a result, both 1D and 2D grating OLEDs gained significant improvement both in luminance and efficiency compared with planar devices. Besides, 2D grating OLED could bring greater enhancement than 1D grating OLED because 2D grating has higher coupling and excitation efficiency for surface plasma and WG mode. This method provides an effective strategy to improve the performance of OLEDs. Wang et al. summarized recent developments of grating structures in optoelectronic devices regarding their typical mechanisms in photon-related devices and their application in many optoelectronic devices. They also have presented how to improve the controllability of fabrication for grating structure and balance the relationship between optical and electrical performance are the bottlenecks that still need to be solved.

Regarding the application examples, several minireviews summarized the recent research activities in developing micro-nanostructures for different optoelectronic devices fields. In the direction of lasers, Chen et al. reviewed the recent advances and existing problems toward microlasers based on organic-conjugated polymers, and the configurations and working mechanism of several typical optical feedback, relevant micro/nano-fabrication strategies and their applications in biological/chemical sensing and organic laser display were also discussed in detail. Periodically arranged photonic crystals fibers could overcome the problems in fiber lasers such as small mode field, low degree of nonlinearity, and non-adjustable dispersion because of their unique nonlinear effects and have been the most important gain medium for high-power ultrashort pulse lasers. Hou et al. discussed recent developments in photonic crystal fiber lasers doped with different ions. They pointed out the major challenge at present is the pulling of photonic crystal fibers and 3D printing microstructured optical fiber process might be an effective approach to solve this problem. In the area of monitoring and sensing, micro-nanostructures are also crucial for optimizing the performance of optoelectronic devices. Yu et al. reviewed latest achievements, the challenges and future prospects of photodetectors with different micro-nanostructures in environmental monitoring, optical communication and electronic information. Meanwhile, Ma et al. gave a minireview of gas sensors based on different micro-nanostructure materials under UV light and visible light activation. They discussed the light activation mechanism and introduced the applications of light-assisted gas sensors with improved properties under light activation.

In addition to the above, in the framework of this special topic, there are also two highly innovative manuscripts on light emitting diodes and carrier dynamics of strong coupling system. Chuai et al. reported the epitaxial growth of Bi_2_Se_3_ thin film by means of molecular beam deposition (MBE) method. The Bi_2_Se_3_ thin film was incorporated into N-Bi_2_Se_3_/P-CuScO_2_ infrared transparent heterojunction diodes because of its remarkable optical transparency in the wide-band infrared region and great *n*-type electrical conductivity. The diodes had an abrupt interface and exhibited rectifying I-V characteristics with the threshold voltage of ∼3.3 V. It provides a promising window electrodes alternatives in infrared detectors, as well as other scenarios in the wide infrared wavelength range. Another research article from Luo et al. investigated a strong coupled system composed of MAPbIxCl3-x perovskite film and Al conical nanopits array by steady-state measurements, which experimentally demonstrated that strong coupling could be achieved with SPs and free charge carriers generated in CH_3_NH_3_PbCl_X_I_3-X_ film. Benefiting from intriguing phenomena originated in strong coupling regime, this work is conducive to develop low-cost nanoplasmonic optoelectronic devices working in a strong coupling regime.

The investigation on transparent conductive electrode is an important complementary part to development of flexible optoelectronic devices in next-generation wearable display and lighting fields (Fan et al., 2019; Kayser and Lipomi, 2019; Wang et al., 2019; Chen et al., 2020). A minireview from Hou et al. firstly systematically analysed advantages and shortcomings of several notable alternative transparent electrodes, and focused on the recent advances in silver nanowire electrodes for flexible organic/perovskite light-emitting diodes regarding the relationship between electrode optimization and device performance. They have presented a perspective on the current challenges and future directions for development of physical mechanisms and encapsulation approach in flexible OLEDs and PeLEDs.

In brief, this Research Topic presents some excellent minireviews and leading-edge researches on the recent advances in micro-nanostructured optoelectronic devices, which shows great potential in military and numerous national economy fields. At this point, we would like to thank all those who have devoted valuable time and effort to this special issue. At the same time, we would also thank the readers for their interests on this special issue. Lastly, we sincerely hope that this Research Topic will inspire the enthusiasm of researchers about micro-nanostructures in optoelectronic devices.

## References

[B1] Bi Y.-G. Feng J. Li Y.-F. Zhang X.-L. Liu Y.-F. Jin Y. (2013). Broadband Light Extraction from White Organic Light-Emitting Devices by Employing Corrugated Metallic Electrodes with Dual Periodicity. Adv. Mater. 25, 6969–6974. 10.1002/adma.201302367 24352984

[B2] Bonaccorso F. Sun Z. Hasan T. Ferrari A. C. (2010). Graphene Photonics and Optoelectronics. Nat. Phot. 4, 611–622. 10.1038/nphoton.2010.186

[B3] Cao J. Yan F. (2021). Recent Progress in Tin-Based Perovskite Solar Cells. Energy Environ. Sci. 14, 1286–1325. 10.1039/d0ee04007j

[B4] Chen X. Xu G. Zeng G. Gu H. Chen H. Xu H. (2020). Realizing Ultrahigh Mechanical Flexibility and >15% Efficiency of Flexible Organic Solar Cells via a “Welding” Flexible Transparent Electrode. Adv. Mater. 32, e1908478. 10.1002/adma.201908478 32103580

[B5] Choi D. H. Nam S. K. Jung K. Moon J. H. (2019). 2D Photonic Crystal Nanodisk Array as Electron Transport Layer for Highly Efficient Perovskite Solar Cells. Nano Energy 56, 365–372. 10.1016/j.nanoen.2018.11.050

[B6] Fan X. Nie W. Tsai H. Wang N. Huang H. Cheng Y. (2019). PEDOT:PSS for Flexible and Stretchable Electronics: Modifications, Strategies, and Applications. Adv. Sci. 6, 1900813. 10.1002/advs.201900813 PMC6774040 31592415

[B7] Feng J. Liu Y.-F. Bi Y.-G. Sun H.-B. (2017). Light Manipulation in Organic Light-Emitting Devices by Integrating Micro/Nano Patterns. Laser Photon. Rev. 11, 1600145. 10.1002/lpor.201600145

[B8] Fu X. Mehta Y. Chen Y. A. Lei L. Zhu L. Barange N. (2021). Directional Polarized Light Emission from Thin‐Film Light‐Emitting Diodes. Adv. Mater. 33, 2006801. 10.1002/adma.202006801 33511698

[B9] Fusella M. A. Saramak R. Bushati R. Menon V. M. Weaver M. S. Thompson N. J. (2020). Plasmonic Enhancement of Stability and Brightness in Organic Light-Emitting Devices. Nature 585, 379–382. 10.1038/s41586-020-2684-z 32939065

[B10] Joo W.-J. Kyoung J. Esfandyarpour M. Lee S.-H. Koo H. Song S. (2020). Metasurface-Driven OLED Displays Beyond 10,000 Pixels Per Inch. Science 370, 459–463. 10.1126/science.abc8530 33093108

[B11] Kayser L. V. Lipomi D. J. (2019). Stretchable Conductive Polymers and Composites Based on PEDOT and PEDOT:PSS. Adv. Mater. 31, 1806133. 10.1002/adma.201806133 PMC6401235 30600559

[B12] Linic S. Chavez S. Elias R. (2021). Flow and Extraction of Energy and Charge Carriers in Hybrid Plasmonic Nanostructures. Nat. Mater. 20, 916–924. 10.1038/s41563-020-00858-4 33398116

[B13] Liu W. Liu M. Liu X. Wang X. Deng H. X. Lei M. (2020). Recent Advances of 2D Materials in Nonlinear Photonics and Fiber Lasers. Adv. Opt. Mater. 8, 1901631. 10.1002/adom.201901631

[B14] Lopez-Sanchez O. Lembke D. Kayci M. Radenovic A. Kis A. (2013). Ultrasensitive Photodetectors Based on Monolayer MoS2. Nat. Nanotech 8, 497–501. 10.1038/nnano.2013.100 23748194

[B15] Mao P. Liu C. Li X. Liu M. Chen Q. Han M. (2021). Single-Step-Fabricated Disordered Metasurfaces for Enhanced Light Extraction from LEDs. Light Sci. Appl. 10, 180. 10.1038/s41377-021-00621-7 34489399 PMC8421350

[B16] Wang C. Xia K. Wang H. Liang X. Yin Z. Zhang Y. (2019). Advanced Carbon for Flexible and Wearable Electronics. Adv. Mater. 31, 1801072. 10.1002/adma.201801072 30300444

[B17] Ye J. Byranvand M. M. Martínez C. O. Hoye R. L. Z. Saliba M. Polavarapu L. (2021). Defect Passivation in Lead‐Halide Perovskite Nanocrystals and Thin Films: Toward Efficient LEDs and Solar Cells. Angew. Chem. Int. Ed. 60, 21636–21660. 10.1002/anie.202102360 PMC8518834 33730428

[B18] Zhou L. Zhou Y. Fan B. L. Nan F. Zhou G. H. Fan Y. Y. (2020). Tailored Polarization Conversion and Light‐Energy Recycling for Highly Linearly Polarized White Organic Light‐Emitting Diodes. Laser Photon. Rev. 14, 1900341. 10.1002/lpor.201900341

